# Supplementary material for: Bacterial lipopolysaccharide modulates immune response in the colorectal tumor microenvironment
Source: NPJ Biofilms Microbiomes. 2023 Aug 23;9:59. doi: 10.1038/s41522-023-00429-w (PMC10447454; doi:10.1038/s41522-023-00429-w)
Supplement: Supplementary file 1 — Supplementary Material [file 41522_2023_429_MOESM1_ESM.pdf]

SUPPLEMENTARY MATERIAL

SUPPLEMENTARY FIGURES

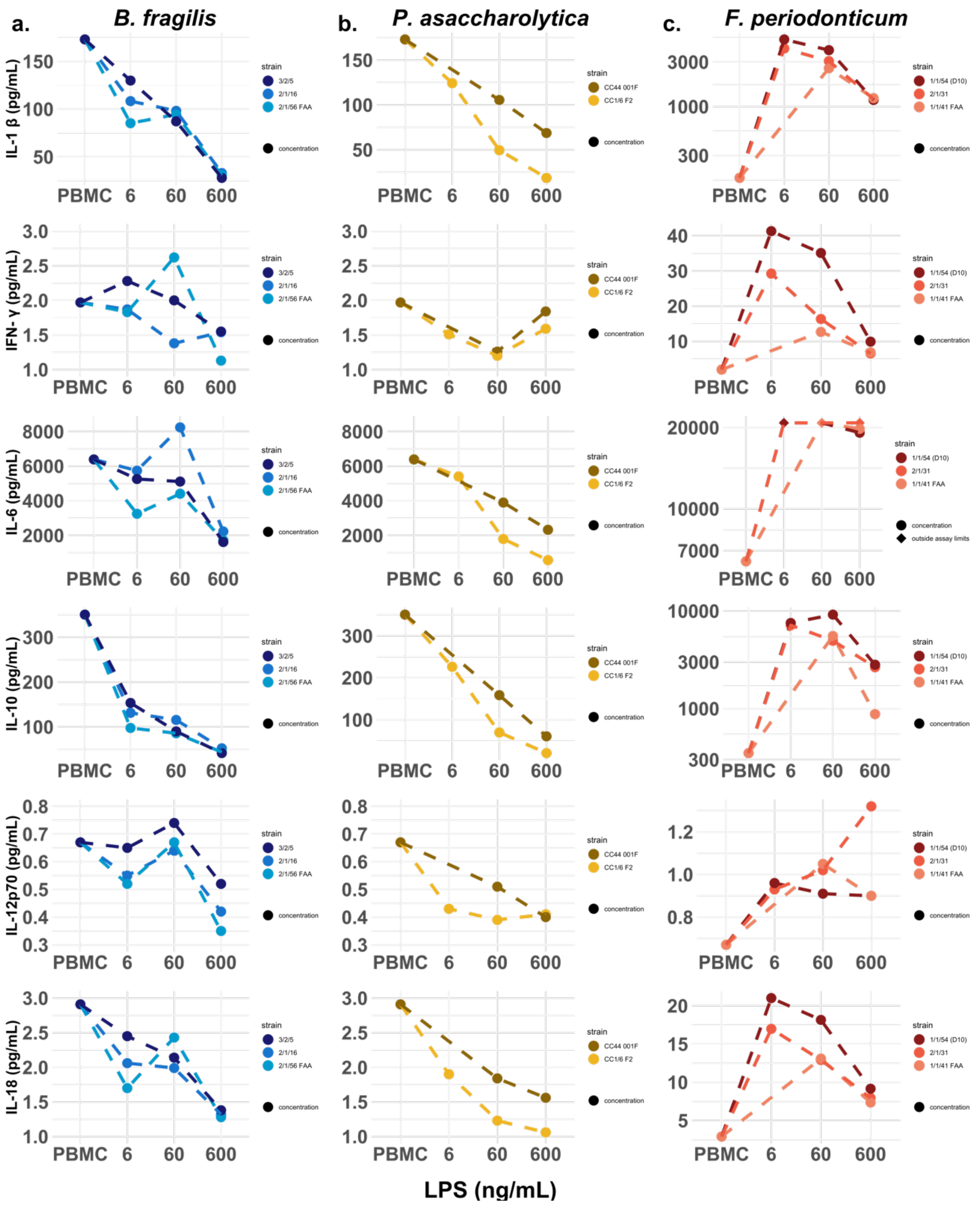

Supplementary Figure 1. Secreted cytokine concentrations (pg/mL) after overnight incubation of PBMCs with different LPS concentrations (6 ng/mL, 60 ng/mL, 600 ng/mL) from different strains of a. *B. fragilis* b. *F. periodonticum*, and c. *P. asaccharolytica* compared to PBMC baseline (no treatment). Dashed lines connect data points from the same strains. Colors differentiate species (*B. fragilis:* blue; *P. asaccharolytica*: yellow; *F. periodonticum:* red) and different shades of a color differentiate the strains of each species. Where values are outside of assay limits, we show the assay limit, and denote the point with '◆'. For *F. periodonticum* plots, IL-1β, IL-6, and IL-10 y-axes are at scale log_{10}.

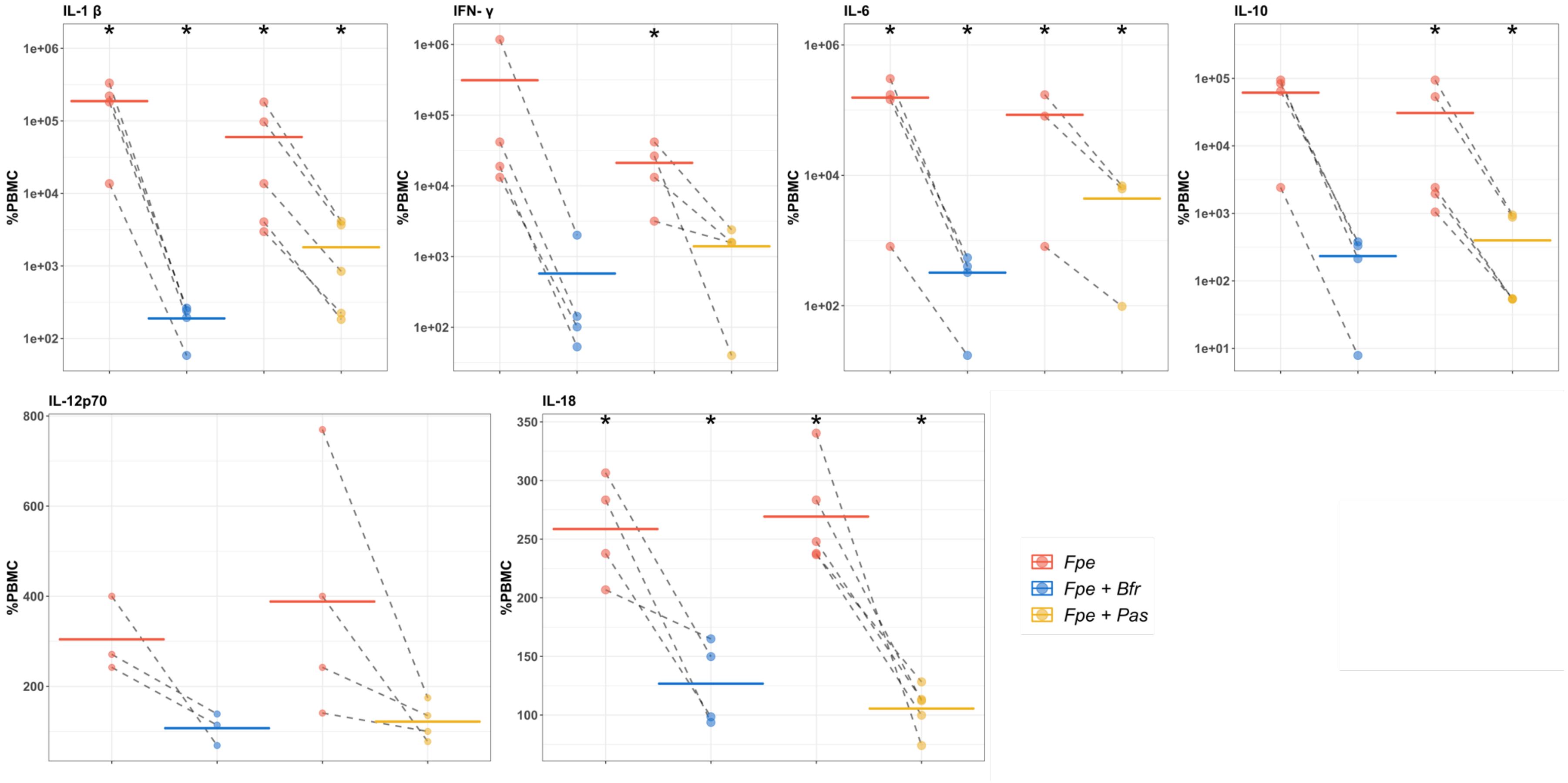

Supplementary Figure 2. Changes in cytokine expression in peripheral blood mononuclear cells (PBMCs) following treatment with *F. periodonticum* alone (red) or in combination with *B. fragilis* (blue) or *P. asaccharolytica* (yellow). We show results for cytokines of interest IL-1β, IFN-γ, IL-6, IL-10, IL-12p70, and IL-18. *F. periodonticum 2/1/31* LPS used was at a concentration of 6 ng/mL. *B. fragilis 2/1/16* LPS and *P. asaccharolytica CC1/6 F2* LPS used were at a concentration of 600ng/mL. Values are shown as percentages of PBMC baseline secretion, which is set at 100%. Dashed lines indicate a single experimental run. Colored, solid horizontal lines represent the means of repeat experiments. Y-axes of IL-1β, IFN-γ, IL-6, and IL-10 are in $\log_{10}$ scale, while Y-axes of IL-12p70 and IL-18 are in linear scale.
Fpe = F. periodonticum (6ng/mL), *Fpe + Bfr = F. periodonticum* (6ng/mL) + *B. fragilis* (600ng/mL), *Fpe + Pas = F. periodonticum* (6 ng/mL) + *P. asaccharolytica* (600 ng/mL); *(*) = Paired student's t-tests p-value < 0.05 (F. periodonticum vs PBMC, F. periodonticum + B. fragilis vs F. periodonticum* alone*, or F. periodonticum + P. asaccharolytica vs F. periodonticum* alone*).*

SUPPLEMENTARY TABLES

Supplementary Table 1. Read count information for each step of the analysis (N=260)			
	Minimum	Maximum	Median
Raw Reads	3518434	18689703	12231622.5
Human Analysis (STAR alignment)[1] - Paired End			
Input	3479791	18670112	12152988
Aligned Uniquely (%)	17.04	93.82	85.84
Human Analysis (STAR alignment)[1] - Single End			
Input	35	5402	2875
Aligned Uniquely (%)	3.7	92.49	84.15
Microbial Analysis[1]			
Input (to Kaiju)	53909	4711734	641559
Identified Species[2] (%)	12.2	65.97	19.02

[1] The trimming and quality control steps provide paired and single end reads which were used to align to the human genome using STAR, separately. It is only after featureCounts that their respective gene counts were combined for subsequent analysis. For microbial analysis, the same was carried out, however Kaiju recommends combining results of paired-end (PE) and single-end (SE) reads immediately, allowing for combined read count information of the PE and SE reads.

[2] Identified species that passed the relative abundance (in percent) cutoff of 0.01%.

Supplementary Table 2. Test of Differences between Cytokine Concentrations Released after PBMC Treatment using *F. periodonticum and B. fragilis* LPS							
**Cytokine**	**Reference Group**	**Group 2**	**N1**	**N2**	**p**	**Cohen's d**	**Magnitude**
**IL-12p70**	PBMC	Fp6	3	3	0.16	-0.72	moderate
	Fp6	Fp6 + Bf600	3	3	0.20	0.62	moderate
**IL-10**	PBMC	Fp6	4	4	0.057	-1.10	large
	Fp6	Fp6+Bf600	4	4	0.055	1.11	large
**IFN-γ**	PBMC	Fp6	4	4	0.09	-0.90	large
	Fp6	Fp6+Bf600	4	4	0.09	0.90	large
**IL-1β**	PBMC	Fp6	4	4	0.041	-1.26	large
	Fp6	Fp6+Bf600	4	4	0.041	1.26	large
**IL-18**	PBMC	Fp6	4	4	0.017	-1.77	large
	Fp6	Fp6+Bf600	4	4	0.05	1.163	large
**IL-6**	PBMC	Fp6	4	4	0.025	-1.52	large
	Fp6	Fp6+Bf600	4	4	0.023	1.57	large
**Legend: Fp6=*F. periodonticum 2/1/31* (6 ng/mL) alone; Fp6+Bf600 =*F. periodonticum 2/1/31* (6 ng/mL) + *B. fragilis 2/1/16* (600 ng/mL) co-incubation; N=number of repeats used in statistical calculations; p=paired t-test p-value; d=cohen's d; Magnitude = interpretation of cohen's d**							

**Supplementary Table 3. Test of Differences between Cytokine Concentrations Released after PBMC Treatment using *F. periodonticum* and *P. asaccharolytica* LPS**							
**Cytokine**	**Reference Group**	**Group 2**	**N1**	**N2**	**p**	**Cohen's d**	**Magnitude**
**IL-12p70**	PBMC	Fp6	4	4	0.06	-1.07	large
	Fp6	Fp6+Pa600	4	4	0.08	0.95	large
**IL-10**	PBMC	Fp6	5	5	0.017	-1.41	large
	Fp6	Fp6+Pa600	5	5	0.016	1.45	large
**IFN- γ**	PBMC	Fp6	4	4	0.035	-1.34	large
	Fp6	Fp6+Pa600	4	4	0.06	1.07	large
**IL-1β**	PBMC	Fp6	5	5	0.010	-1.63	large
	Fp6	Fp6+Pa600	5	5	0.011	1.61	large
**IL-18**	PBMC	Fp6	5	5	0.02	-1.35	large
	Fp6	Fp6+Pa600	5	5	0.021	1.33	large
**IL-6**	PBMC	Fp6	3	3	0.017	-2.52	large
	Fp6	Fp6+Pa600	3	3	0.015	2.69	large
**Legend: Fp6=*F. periodonticum 2/1/31* (6 ng/mL) alone; Fp6+Pa600=*F. periodonticum 2/1/31* (6 ng/mL) + *P. asaccharolytica CC1/6 F2*(600 ng/mL) co-incubation; N=number of repeats used in statistical calculations; p=paired t-test p-value; d=cohen’s d; Magnitude = interpretation of cohen's d**							
