# Supplementary material for: Bacterial lipopolysaccharide modulates immune response in the colorectal tumor microenvironment
Source: NPJ Biofilms Microbiomes. 2023 Aug 23;9:59. doi: 10.1038/s41522-023-00429-w (PMC10447454; doi:10.1038/s41522-023-00429-w)
Supplement: Supplementary file 2 — Reporting Summary [file 41522_2023_429_MOESM2_ESM.pdf]

nature portfolio

Corresponding author(s): Sulit, Arielle Kae

Last updated by author(s): Apr 4, 2023

Reporting Summary

Nature Portfolio wishes to improve the reproducibility of the work that we publish. This form provides structure for consistency and transparency in reporting. For further information on Nature Portfolio policies, see our Editorial Policies and the Editorial Policy Checklist.

Statistics

For all statistical analyses, confirm that the following items are present in the figure legend, table legend, main text, or Methods section.

n/a	Confirmed	
☐	☒	The exact sample size (n) for each experimental group/condition, given as a discrete number and unit of measurement
☐	☒	A statement on whether measurements were taken from distinct samples or whether the same sample was measured repeatedly
☐	☒	The statistical test(s) used AND whether they are one- or two-sided<br>*Only common tests should be described solely by name; describe more complex techniques in the Methods section.*
☐	☒	A description of all covariates tested
☒	☐	A description of any assumptions or corrections, such as tests of normality and adjustment for multiple comparisons
☒	☐	A full description of the statistical parameters including central tendency (e.g. means) or other basic estimates (e.g. regression coefficient) AND variation (e.g. standard deviation) or associated estimates of uncertainty (e.g. confidence intervals)
☐	☒	For null hypothesis testing, the test statistic (e.g. F, t, r) with confidence intervals, effect sizes, degrees of freedom and P value noted<br>*Give P values as exact values whenever suitable.*
☒	☐	For Bayesian analysis, information on the choice of priors and Markov chain Monte Carlo settings
☒	☐	For hierarchical and complex designs, identification of the appropriate level for tests and full reporting of outcomes
☐	☒	Estimates of effect sizes (e.g. Cohen's d, Pearson's r), indicating how they were calculated

Our web collection on statistics for biologists contains articles on many of the points above.

Software and code

Policy information about availability of computer code

Data collection	no software was used for data collection
Data analysis	Bioinformatics for gene and microbial profiling was done using MetaFunc (10.1017/gmb.2022.12) and subsequent analyses are found in gitlab repository (https://gitlab.com/alsulit08/2021_uoc_massey_lps-crc/-/tree/master)

For manuscripts utilizing custom algorithms or software that are central to the research but not yet described in published literature, software must be made available to editors and reviewers. We strongly encourage code deposition in a community repository (e.g. GitHub). See the Nature Portfolio guidelines for submitting code & software for further information.

Data

Policy information about availability of data

All manuscripts must include a data availability statement. This statement should provide the following information, where applicable:

- Accession codes, unique identifiers, or web links for publicly available datasets
- A description of any restrictions on data availability
- For clinical datasets or third party data, please ensure that the statement adheres to our policy

Sequencing data can be found under Bioproject ID PRJNA788974 in the NCBI SRA database. Details for bioinformatics and statistical analyses may be found at https://gitlab.com/alsulit08/2021_uoc_massey_lps-crc. Limited patient metadata may be provided upon reasonable request to authors.

Human research participants

Policy information about studies involving human research participants and Sex and Gender in Research.

Reporting on sex and gender	We only collect information on participant sex, which is based on self-reporting. Sex and gender were not specifically considered in designing the study, and results of this paper is not sex-specific. De-identified, individual sex information may be found with the sequencing data in the NCBI SRA with Bioproject ID PRJNA788974. A total of 121 males, and 139 females were included in the study.
Population characteristics	Patients that will undergo surgical resection due to Colorectal Cancer are included in the study. There are no age restrictions. Exclusion criteria include prior therapy, and diagnosis of hereditary non-polyposis colorectal cancer (HNPCC) or familial adenomatous polyposis (FAP).
Recruitment	Recruited with informed consent and ethical approval through an institutional tissue bank
Ethics oversight	University of Otago Human Ethics Committee

Note that full information on the approval of the study protocol must also be provided in the manuscript.

Field-specific reporting

Please select the one below that is the best fit for your research. If you are not sure, read the appropriate sections before making your selection.

☒ Life sciences ☐ Behavioural & social sciences ☐ Ecological, evolutionary & environmental sciences

For a reference copy of the document with all sections, see nature.com/documents/nr-reporting-summary-flat.pdf

Life sciences study design

All studies must disclose on these points even when the disclosure is negative.

Sample size	Samples used were based on available samples and is an observational study
Data exclusions	Samples that were not included in any consensus molecular subtypes were excluded.
Replication	This study is an observational study and replication couldn't be performed.
Randomization	This study is an observational study and no pre-determined groups were used. As such, no randomization was required.
Blinding	This study is an observational study and no pre-determined groups were needed. Blinding was therefore not required.

Reporting for specific materials, systems and methods

We require information from authors about some types of materials, experimental systems and methods used in many studies. Here, indicate whether each material, system or method listed is relevant to your study. If you are not sure if a list item applies to your research, read the appropriate section before selecting a response.

Materials & experimental systems

n/a	Involved in the study	
☒	☐	Antibodies
☒	☐	Eukaryotic cell lines
☒	☐	Palaeontology and archaeology
☒	☐	Animals and other organisms
☒	☐	Clinical data
☒	☐	Dual use research of concern

Methods

n/a	Involved in the study	
☒	☐	ChIP-seq
☒	☐	Flow cytometry
☒	☐	MRI-based neuroimaging
